# Improving Safety Performance of Construction Workers through Learning from Incidents

**DOI:** 10.3390/ijerph20054570

**Published:** 2023-03-04

**Authors:** Albert P. C. Chan, Junfeng Guan, Tracy N. Y. Choi, Yang Yang, Guangdong Wu, Edmond Lam

**Affiliations:** 1 Shenzhen Research Institute of the Hong Kong Polytechnic University, Shenzhen 518057, China; 2 Department of Building and Real Estate, The Hong Kong Polytechnic University, Hung Hom, Kowloon, Hong Kong 999077, China; 3 School of Public Policy and Administration, Chongqing University, Chongqing 400044, China; 4 College of Professional and Continuing Education, The Hong Kong Polytechnic University, Hung Hom, Kowloon, Hong Kong 999077, China

**Keywords:** safety learning, learning from incidents, Bayesian network, construction industry, safety performance

## Abstract

Learning from incidents (LFI) is a process to seek, analyse, and disseminate the severity and causes of incidents, and take corrective measures to prevent the recurrence of similar events. However, the effects of LFI on the learner’s safety performance remain unexplored. This study aimed to identify the effects of the major LFI factors on the safety performance of workers. A questionnaire survey was administered among 210 construction workers in China. A factor analysis was conducted to reveal the underlying LFI factors. A stepwise multiple linear regression was performed to analyse the relationship between the underlying LFI factors and safety performance. A Bayesian Network (BN) was further modelled to identify the probabilistic relational network between the underlying LFI factors and safety performance. The results of BN modelling showed that all the underlying factors were important to improve the safety performance of construction workers. Additionally, sensitivity analysis revealed that the two underlying factors—information sharing and utilization and management commitment—had the largest effects on improving workers’ safety performance. The proposed BN also helped find out the most efficient strategy to improve workers’ safety performance. This research may serve as a useful guide for better implementation of LFI practices in the construction sector.

## 1. Introduction

The construction industry is one of the highest accident rates and labor-intensive industries [1]. Safety is the priority for all construction projects. Despite considerable efforts by government, industry, and academia to improve the safety performance of construction projects [2], occupational incident rates among construction workers remain high [3,4]. The temporary nature and specificity of the work sites demonstrate how the construction industry behaves differently and has unique characteristics [5]. Companies, workers, technicians, and others gather at each site at a certain time and place to construct something new and distinctive with a variety of processes. Each process occurs and possesses impact on one another, each with different dangers. In addition to the distinct identities of the cooperating companies, each site has its own character, organizational structure, and material and human resources. Processes, machinery, resources, and workmanship can vary greatly depending on the construction site. This high level of variability creates dangers and safety protective measures that are unique to each site, and is likely to cause issues with reducing accident rates, which are among the highest and most common in the world in the construction sector [6,7,8]. Therefore, a wide range of factors, including the environment, the surrounding area, structural issues, and cultural aspects, have an impact on construction sites. This argument was emphasized by Peckitt et al. [9] by pointing out the features of the construction industries that have a negative impact on the performance of the industry’s safety measures, and the difficulties of eliminating risks and hazards in construction. Additionally, in order to ensure safety and make the industry more efficient in this regard, an analysis that takes into account LFI factors in construction that allows for the development of proactive health and safety interventions is necessary.

Poor safety performance is a major problem in the construction industry worldwide. In China, construction incidents typically account for more than one-third of all industrial incidents each year [10,11]. Some previous studies investigated the causes of accidents in different industries. For example, Carrillo-Castrillo [12] examined the root causes of the most common accidents in the manufacturing sector, and prioritized the most effective preventive measures. The specific causation pattern of each accident mechanism was identified. It can be utilized to determine efficient preventive measures and avoid the most common causes of each accident mechanism. Winge et al. [13] reviewed 176 relatively severe construction accidents and revealed key causal connections between accident factors. The analyses discovered seven causal factors that were consistently associated with worker actions, such as immediate supervision and local hazards. There were also strong links noticed between risk management and immediate supervision, as well as risk management and worker actions. The empirical factors impacting unsafe behaviors and accidents on construction sites were studied by Khosravi et al. [14]. In the UK, approximately one-third of all occupational deaths were from the construction industry [15]. Moreover, the industry underperformed in terms of frequent employee injuries and poor health conditions [15]. The number of incidents in the Norwegian construction industry was much higher than that of other industries [4]. The fatality rate of construction workers was significantly higher than that of other industries in Australia [3]. The incident data indicated that most of the workers’ injuries and deaths were attributed to similar incidents, such as vehicle collisions, being hit by falling objects, and falling from height [3,16]. The frequent occurrence of similar incidents in the construction industry may imply that learning from incidents (LFI) is far from good practice in the construction industry [17,18,19]. LFI is defined as a process through which “employees and the organization as a whole seek to understand any negative safety events that have taken place in order to prevent similar future events” [20]. LFI is an effective means to prevent the recurrence of similar incidents. Therefore, it is urgent to deepen the understanding of researchers and practitioners on how to learn from past incidents in the construction industry. Considered as a whole, most of the previous studies were focused on specific aspects of construction safety, including research trends, safety information flow, and innovative technology applications in construction safety [21], but very few studies have been conducted on the subject of learning from incidents in the construction sector. From the existing LFI-related research, it is widely accepted that LFI is critical to preventing the recurrence of incidents (e.g., [20,22]). 

However, empirical studies establishing the relationship between LFI factors and safety performance are still lacking. While LFI has been recognized as an important means of incident prevention, its impact on safety performance remains unexplored. The current study aimed to fill the research gap by determining the relationship between LFI factors and safety performance. By drawing upon current literature, the study proposed hypotheses about the impact of major LFI factors on the safety performance of construction workers. Then, factor analysis was applied to identify the underlying dimensions of the LFI factors. The stepwise multiple linear regression was performed to determine the effects of the underlying LFI factors on the safety performance of construction workers. Based on the causal-effect relationship between the LFI factors and safety performance, Bayesian Network (BN) was further established to present the probabilistic relationship between LFI factors and safety performance. Finally, the contributions, limitations, and implications of the BN model were discussed. The results of this study are expected to contribute to the existing body of knowledge in the field of learning from construction incidents as a theoretical reference for building LFI systems in construction organizations. The proposed model could be used as a decision-making tool for construction practitioners to compare various LFI intervention strategies and determine the best among them.

The rest of this paper is organized as follows. Section 2 reviews and categorizes LFI factors. Section 3 presents the overall research methodology and describes the data collection process. Section 4 and Section 5 reports the main findings resulting from BN inference and discusses them in detail. Section 7 summarizes the paper and draws some conclusions about future research directions.

## 2. Literature Review and Hypotheses Development

### 2.1. Management Commitment

Conscious effort in fostering a learning culture is crucial to encourage organizational members to draw from lessons on past incidents [23,24]. Creating a just culture is essential for enabling learning, where there is an open and non-punitive environment [25], incidents are recognized as great learning opportunities [26], employees are motivated and sometimes rewarded in recognition of sharing of safety/incident information [27], and management shows commitment to safety through leadership by example [28]. 

Management commitment in the context of organizational learning capability refers to the management’s ability to understand and convey the importance of learning. They should start driving the change process and, when necessary, abandon old beliefs [29]. The commitment of management to safety has been identified as a key success factor in improving safety performance [30]. A key component of the safety climate that has been found to be important for safety performance is high management commitment to safety [31,32,33]. To foster a culture where mistakes and failures can be learned from in the past and present without fear of being blamed, and to encourage behavioral change, senior management support is essential [34,35,36]. The safety commitment of senior management is essential for organizational safety initiatives to be successful [37]. 

Organizational learning requires an open mentality towards mistakes, incidents, and safety. Openness relates to the ability to question existing knowledge. It is possible to make mistakes when exploring new options and ideas in the search for innovations, which allows for the learning of failures. Management openness, including a desire to openly and truthfully exchange ideas and information, is widely mentioned as a feature or dimension of management quality required for the establishment of trust in management [38]. To be effective, communication should take place not just between management and employees, but also amongst employees in an open and mutually trusting atmosphere. High management commitment, according to Hofmann and Stetzer [39], promotes open communication regarding safety, and sends a clear message about how important safety is to the company or organization. In order to foster cooperation and integration, as well as information exchange, communication is crucial for promoting safety [40]. The employer, worker, and subcontractor representatives are frequently present on a safety committee. This promotes conversation between the parties, enhances trust and communication, and allows each party’s expertise to be utilized [41]. The findings of Törner and Pousette’s [42] study showed that the effects of management attitudes and individual attitudes on safety performance interact and reinforce one another. According to the research, a construction company’s management commitment to safety encourages the growth of open and trustworthy relationships both inside and outside the organization, including with other construction contractors and organization. The safety managers and particularly the project manager had a wealth of management experience in safety-related issues and gave workers useful information. To improve workplace safety, it is important for managers and supervisors to motivate workers to share their safety experiences [43]. According to Aksorn and Hadikusumo [44], management support has the greatest impact on safety. In light of their previous experience, active roles of management staff will show a sincere concern for the safety of employees, which in turn will contribute to foster a positive attitude among employees. 

The establishment of a management hierarchy that is in charge of making decisions and establishing specific milestones is necessary in the dynamic environment of the construction industry. When unforeseen changes do occur, it can be difficult and challenging to plan, organize, inspire, direct, and control construction [45]. The environments of construction projects, both internal and external, are comparatively unstable. The management and organization of a project may be significantly impacted by changes that take place during its development. As a result, management and supervisory staff should respond appropriately to change (i.e., new risks are being discovered, hazardous events are occurring) and put the necessary corrective measures in place immediately. An efficient information system should be created to enhance the integration of various project participants. On-the-job training can improve a worker’s performance and quality. Similarly, by implementing training and development programs in areas such as leadership, decision-making, and relationships between employees and supervisors, managerial effectiveness can be increased [45].

### 2.2. Learning Content

Safety can be defined as a set of practices comprised of competencies acquired through engagement and participation in daily activities [46]. For the construction industry to maintain and improve its safety performance, organizational learning is essential. According to statistical reports released by the Ministry of Housing and Urban-Rural Development (MOHURD) of China [47], there were 1567 construction accident cases in China from 2018 to 2020. The largest number of construction accidents were in the “fall from a high place” category, accounting for over 50% of all construction accidents. Additionally, cases involved being struck by an object, collapses, and injuries from lifting, accounting for nearly 35% of the total number of construction accidents. Other accident types consisted of mechanical injuries, fires and explosions, poisoning and asphyxiation, vehicle injuries, drowning, and electric shocks. The greatest number of fall from height accidents in construction occurred along openings and edges, followed by scaffolds and tower cranes. Since these types of accidents occur frequently, employers and employees should conduct in-depth analysis and investigations on these accidents to improve learning effectiveness. Although the value of organizational learning and knowledge management has been widely acknowledged, critics contend that current organizational practices ignore the challenges and complexities involved in the process of knowledge creation and learning [48]. According to Cooke and Rohleder [49], an organization with a higher capacity for learning from incidents will encounter fewer incidents than one with a lower learning capacity.

The organizational learning literature [50,51] suggested that more efficient organizational learning from incidents lowers the possibility of incident occurrence and severity incidents. The risk of unfavorable events may be reduced by improving the organizational learning system. This encourages us to measure organizational capabilities for incident-based learning. The investigation and learning aspects of the system had the biggest impact on the ability of the entire organization to learn from incidents, according to Cooke and Rohleder’s [49] research findings. The components of the learning system include incident identification and response, reporting, investigation, causal structure determination, recommendation making, sharing and recalling incident learning, and putting corrective measures into practice.

Stemn et al. [28] pointed out that it is beneficial to learn from both external sources and prior internal experience. Employees who have the chance to learn from incidents will need to share their insights more widely within the company or organization and within the industry. Only when there are learning systems in place allows organizations to learn from incidents. An incident learning system is a set of organizational capabilities and processes used to understand, collect, and retrieve practical knowledge from all types of incidents, including near misses, and to implement the lessons so that an organization’s safety performance can be improved [48]. A channel for sharing lessons is necessary for organizations to learn from one another. Construction and safety professionals can gather at conferences, seminars, or workshops to discuss significant mistakes and unforeseen incidents with the aim of learning [24]. Stemn et al. [28] identified this as an important indicator in the current context, where construction practitioners should focus on recognizing how organizations learn from the mistakes of others (both within and across industries), how extensively lessons are discussed, and the information processes used to convey lessons at various levels.

Previous studies on safety recognized the importance of learning from past near misses and accidents in order to prevent future ones [52,53,54]. Continuous monitoring and systematic analyses of issues, deviations, incidents, and near misses reveal organizational weaknesses and latent failures [27]. The objective of higher reliability and safety should be achieved by using incident analysis results to create new knowledge, new regulations, and new practices [50]. It is impossible to completely prevent worker mistakes or unsafe working conditions in construction projects. However, a proactive and successful approach to safety management can be developed by construction project managers with the aid of an early warning system. Near misses that happen while project workers are working should be encouraged to be reported [55].

Accidents or near-accidents on construction sites help workers understand the value of safety and reinforce safe work practices. Choudhry & Fang [43] suggested that toolbox talks could be used to help employees share these near misses more effectively. Additionally, management can influence employee behavior changes for the better in terms of safety through training, increased communication, and rules and regulations. Safety personnel can take the lead in reporting near misses by workers at daily meetings on safety. The meeting included an explanation of the near-miss details and the fact that any disciplinary action resulting from such communication would be avoided [56]. The workers were also urged to report any safety violations they thought were important. The analysis of near misses includes a risk assessment of each event, taking into account its severity and likelihood of happening. This assessment is required to prioritize implementing preventive and corrective measures, and to justify further investigation into the events with the highest risk [56]. With a focus on high potential incidents, near-miss analysis has the potential to be a great addition to the incident data set [57]. Cooper [58] proposed that a near-miss reporting sub-system be developed as well. Through this sub-system, any unusual occurrences that could cause an accident can be reported. To reduce safety risks, additional research can be conducted and preventative measures should be taken [59].

### 2.3. Information Sharing and Utilization

Lessons learned should be stored, retrieved, and transferred to develop organizational memory and apply the knowledge learned. Nevertheless, the dissemination of incident information is viewed as a weak point during the learning process [60]. The lessons should be distributed broadly and documented in a way which enables all workforce to retrieve past lessons, and utilize them when needed [28]. Through interactive communication and discussion, employees can understand what happened and what should be conducted to avoid similar incidents from happening again. The formal and informal ways of communication can be adopted to enhance the exchange of incident information [22,28]. Ineffective implementation of remedial actions could contribute to a failure in learning [51]. Actions that are not performed well can be attributed to a lack of clear ownership, poor motivation in performing actions, fear of extra work [51,61], and a lack of financial resources [62]. For effective learning, the investigation and analysis of an incident should be followed by effective and prompt implementation of corrective actions [28,60,63]. The effectiveness of recommended corrective actions should be periodically evaluated to manage potential risks associated with those actions [28,49].

In the age of big data, information is a key component that significantly affects construction safety. Luo and Wu [64] pointed out that the primary cause of accidents is the loss or asymmetry of safety information. Huang et al. [65] showed that information is the carrier of accident chain progression and information flow (IF) is the link between the organizational, behavioral, and perceptual components of accidents. The smoothness of safety information flow will depend on a wide range of variables due to its complicated structure. Accidents are typically caused by improper safety information supply, faulty safety information feedback, and incorrect safety information cognition. According to Wang and Wu [66], one of the main causes of fatal human accidents is the inadequate provision of safety information, which results in a lack of safety information. To minimize hazardous accident occurrences and risk in the construction business sector, effective knowledge and information sharing on safety is becoming extremely important [65]. An efficient safety and health communication system is required to identify, evaluate, and assess safety risks in order to achieve better safety performance. To share pertinent incident information and develop proactive accident prevention strategies during the construction process, effective communication channels are crucial.

The dissemination of the results from incident investigations forms the foundation of many organizations’ initiatives to help employees learn from incidents [59,60]. People should actively engage with incident information and, where necessary, change practice in order to learn, rather than just passively receiving it [67]. Moving individual employees toward actively interacting with incident information is problematic for these two reasons [68]. First, rather than having employees actively participate in incident knowledge, many LFI activities place more of an emphasis on having them receive and read incident information. Second, there are not enough opportunities during the workday for employees to consider and interpret incident data in light of their own job responsibilities.

Previous research indicated that front-line supervisors have a significant impact on safety [69,70]. It has long been argued that the supervisor, or front-line manager, is a key player in accident prevention because they have daily contact with staff and the chance to control the unsafe situations and behaviors contributing to accidents [71]. According to the literature, effective supervisory behavior includes a number of critical elements, including supervisors’ attitudes and approaches to safety and training, their level of interaction with their staff, their thoroughness, and willingness to learn from accident investigations, as well as how much time they spend with them [71]. It is very important for the supervisor to assist the workers in providing appropriate safety information and incident related information.

Numerous studies have found that the majority of accidents and injuries are caused by unsafe work practices by employees rather than unsafe working conditions [72,73]. Almost all of the interviewees of Choudhry and Fang’s [43] research believed that the main cause of unsafe behaviors on-site was a lack of skill. To improve the worker’s skill and safety knowledge, it is imperative to provide pertinent incident information. Accidents can be minimized in an environment that promotes safe communication, according to Hofmann and Stetzer [39]. In other words, employees are less likely to have accidents than those who do not actively share information about accidents in their group.

Information about an accident at a construction site will spread among the workers. The rate of diffusion is based on how well and/or frequently workers communicate about the accidents. If workers recognize the need to alter their risk perception, accident information can have a significant impact on their safety behavior and awareness. A key factor in determining whether the workers’ attitudes toward safety change is how sensitively they respond to accidents during this process [74]. Sharing accident data among co-workers can help reduce the number of accidents because workers frequently overestimate their ability to manage accident risks. When employees are involved in accidents directly or indirectly, their attitudes toward safety can be changed as a result. Workers will benefit from being immersed in accidents as part of the accident reporting process because they assess the risk of accidents based on how likely it is that it will happen to them [74]. It will be effective to provide as much information about the accident as possible, or to give workers the opportunity to indirectly experience the accident via audiovisual materials. It is crucial for the employees to recognize any unsafe behaviors at the workplace based on information from incidents, and to change their unsafe behaviors accordingly.

### 2.4. Implementation of Lessons

Lessons are then communicated and disseminated to stimulate the learning participants to learn. Dissemination of incident information does not, however, always lead to a change in unsafe behavior or practices [75]. Hence, the lessons learned should be translated into actions that are well implemented by addressing when to perform, what resources to allocate, and who to perform actions [51]. Finally, the effectiveness of the actions implemented should be monitored and evaluated to verify whether they bring about behavioral/organizational change and improve safety [23,51]. Most accidents can be prevented by using the previously existing data because they are remarkably similar to past occurrences [76]. As learning from the incident cycle is insufficient to prevent accidents, construction mishaps continue to occur [77]. Some repeated accident cases in Kai Tak Development Project are summarized in Table 1 to show how the knowledge can be extracted in the incident learning process. The cases were analyzed based on an actual fatal incident investigated by the Coroner’s Court in Hong Kong. In Case 1, the accident occurred while the deceased was seated on the stepladder’s third-from-top rung. Riding on the stepladder was an unsafe act. The stepladder’s rungs were too narrow to give the worker a secure platform. There were no rigid ladder locks to keep the ladder in place. To reduce the spreading angle between the ladder legs, only a nylon rope was attached at the top sixth pair of opposite ladder rungs. When performing the conduit wiring work in such hazardous circumstances, the decedent was unable to maintain his balance. He consequently dropped off the stepladder onto the ground. Although the decedent was wearing a safety belt, the investigation report showed that there was no proper anchorage point or independent lifeline to secure the safety belt. The failure to provide and maintain a safe system for conduit wiring work, as well as the use of improper and unsafe means of support, were the underlying accident causes. The employer failed to provide information, instruction, training and, supervision to workers. The investigation revealed that there were no explicit measures in place to prevent the accident from happening. If a safe system of work was conducted, suitable scaffolds or working platform were provided, and necessary information and supervision were provided for the workers, the accident could have been prevented. Therefore, an effective LFI process would be essential as the guidelines for the industrial practitioners to take appropriate safety measures to prevent the recurrence of similar events.

According to Cannon and Edmondson [24], assisting employees in identifying failure requires a proactive and skilled investigation, as human intuition frequently falls short of extracting the most important lessons from failure. As a result, specialized training is an essential tool for incident learning. Such training could be formal and structured (such as through courses or training), or informal (e.g., on-the-job learning) [22]. Proactive strategies can be the most appropriate method to minimize construction incidents. Serious incidents can also be decreased by educating and training construction workers based on incident data [78]. Furthermore, the development of safety training sessions for employees, as well as seminars and talks focusing on work connected to the incident, may have a positive effect on employee behavior, resulting in a decrease in incidents [79,80]. Incident information is vital for training purposes. It can improve workers’ non-technical skills such as teamwork, communication, prioritization, leadership, and situation awareness in addition to their technical skills.

Slvia et al. [81] noted that, while many organizations have policies and procedures in place to report accidents and gather data on them, some still do not take full advantage of their opportunities to learn from workplace accidents. One of the four main processes in formal organizational learning from incidents is having clear rules and communication channels for staff to share pertinent safety information [81]. In general, learning and decision-making processes that necessarily require accurate and unbiased information are necessary to prevent workplace accidents. Effective sharing of incident data should be a crucial step in a well-established continuous learning process for it to be effective [82]. Encouragement of employees to share incident information with their co-workers is extremely important. Diffusion, discussion, training, and change are the four types of interventions proposed by Silva and Lima [83] to support learning from accidents. The initial step, “Diffusion,” refers to the communication of pertinent incident data. Discussion in the second step refers to an exchange of opinions on the accident-related information. The third step, “Training,” involves using incident information to enhance worker safety performance through safety training. The final step after an accident is “Change,” which denotes a change in the organization (e.g., updating rules, technologies, supervision, and inspection systems, etc.). Dissemination is one of the five key learning components in accident learning phase, as identified by Littlejohn et al. [22]. It involves information exchange between co-workers, as well as sharing updates and findings from incident investigations. Besides, organizations should put in place a reward system that motivates incident reporting and the execution of corrective measures. These two crucial steps, which open and close the incident learning cycle, must be properly carried out for safety improvement in a company to occur.

### 2.5. Management Commitment on Learning from Incidents/Information Sharing and Utilization/Learning Content/Implementation of Lessons

#### 2.5.1. Learning from Incidents

The ability of an organization to properly attribute incident causes and draw conclusions from safety incidents has been linked to management commitment [39,84]. In order to prevent workplace accidents, management at all levels must fully commit to applying the lessons learned from incidents. According to the research by Hallowell and Gambatese [85], top management commitment is the most efficient safety management component related to accident learning, analyzing, and emergency management planning. According to Gunningham and Sinclair’s [86] research, an effective safety management system requires that informal factors such as commitment and trust support the formal system. The most crucial element for a successful safety program was management commitment and involvement in safety [87]. Higher capability in incident investigation, analysis, and learning indicates that the company is more committed to safety management and has more resources available. The research of Goh and Chua [88] indicated that projects with stronger management commitment to safety are generally less likely to experience accidents, and even when they do, the severity of those accidents should be reduced. Thus, one of the crucial elements that contribute to accident learning is management commitment.

#### 2.5.2. Information Sharing and Utilization

The commitment of top management to fully share and use accident information is crucial. To learn from accidents, it would be important to implement a program to undertake incident reporting, analysis, and information sharing and utilization. For the program to be successful, extra resources must be allocated. It makes sense that, if upper management is not committed to the program, there will likely not be enough financial support allocated to ensure the success of the accident learning program [89]. Effective communication, accident information transfer, and utilization between management and employees will result in higher safety standards and improved policy implementation [90]. The management’s commitment to safety has been identified as a determinant of the various modes of communication and accident information transfer to all levels of the construction project [91]. Thus, the sharing and utilization of accident information in an organization is dependent on the management and workers’ commitment to safety. As shown in some studies, management commitment to safety is one of the key elements in improving employee sharing of safety information [92,93,94]. The management team and supervisor promote open communication and the sharing of accident information. The management level of the hierarchy is the most crucial one for ensuring that accident data can be used and shared effectively. It may not be possible to improve safety and health on construction sites without the management’s support and their strong commitment to safety [95].

#### 2.5.3. Learning Content

Without actually experiencing an incident, learning from near misses can increase organizational productivity and improve safety [20]. Strong management commitment is essential to support the entire learning process. For both employees and the organization, determining the cause of a near miss, figuring out what went wrong, and responding to a near miss are critical tasks [96]. Near-miss reports can present a valuable opportunity for organizations managing accident learning systems to learn and enhance safety. By analyzing incident reports, organizations can learn more about how to improve safety [49]. This, in turn, strengthens the organization’s ability to address persistent unsafe conditions, and influences management’s commitment to safety improvement. According to Jiang et al. [97], management commitment has a positive impact on employee behavior, which can lead to both an increase in reported incidents and a decrease in the actual number of incidents. To identify near misses and take immediate corrective action to reduce future risks, corporate management needs to demonstrate a commitment to safety support [98]. Therefore, reporting near misses is an essential learning tool and metric for any safety management system [99,100]. In contrast, a lack of an accident learning system in a company with poor management commitment implies that near misses or incidents are more probable to occur that cause harm and raise costs [101].

#### 2.5.4. Implementation of Lessons

In order to ensure the execution of the strategic actions determined by the organization’s top management, management commitment is essential. This commitment takes into account the need to disseminate information in a rapid and effective manner, as well as guaranteeing the appropriateness of the information [102,103]. A prompt and thorough investigation, as well as the dissemination and implementation of corrective actions, are necessary to prevent incident recurrence [104]. Management commitment strengthened the implementation of the safety program through the visible support of the top management, which also included motivating all employees to succeed through teamwork and setting realistic safety goals [105]. The management should demonstrate their commitment to safety by putting in place a sound safety management system, including incident and near-miss reporting systems and accident incident information dissemination systems. Allocating sufficient resources is important to ensure that workers and other people who may be exposed to risks are provided of all accident-related details [106]. All learning from incidents factors were summarized in Table 2.

### 2.6. Hypotheses Development

This paper hypotheses that safety performance of construction workers could be improved through learning from incidents; that is, workers who can effectively draw lessons from construction incidents have better safety performance.

**H1:**  *Incident information shared and utilized by workers can improve their safety performance*.

**H2:**  *Management commitment on learning from incidents, better safety performance of construction workers*.

**H3:**  *Follow-up actions performed by workers can improve their safety performance*.

**H4:**  *More comprehensive lessons learned by workers, better safety performance they have*.

## 3. Methodology

The following sections introduce the overall research method shown in Table 3 and describe the development process of the Bayesian Network (BN).

### 3.1. Survey Questionnaire

This study forms a part of a large-scale questionnaire survey that aims to investigate the level of incident learning among frontline workers and management staff in Chinese construction projects. The overall questionnaire consists of three sections. The first section contains the measurements of the LFI factors. Respondents were asked to rank each parameter with a Likert-scale score from 1 (strongly disagree) to 5 (strongly agree) [22]. The second section is to measure the safety performance. The last section is about the demographic information of respondents. The questionnaires were peer-reviewed by academics and professionals in the construction industry. A total of 11 reviewers were invited to provide feedback to the questionnaire designed with the aim of ensuring that the statements of the questions could be easily understood by frontline workers. These reviewers included six scholars in Australia, Hong Kong, and Mainland China, and five construction professionals engaged from Chinese construction projects. These reviewers are all proficient in English and Chinese, and they were further requested to advise the consistency of the questionnaire in bilingual language. The feedback of all reviewers was collected from April to June 2021 to refine the questionnaires. The questionnaires were distributed to contractors in Hong Kong and Mainland China. Electronic questionnaires were conducted using a QR code link. In this paper, 15 questions related to LFI factors and 6 questions related to frontline workers’ behavioral safety performance were used for statistical analysis. A total of 210 responses were gathered. Table 4 shows the demographic information of the respondents.

### 3.2. LFI Factors Identification

#### 3.2.1. Exploratory Factor Analysis 

A total of 15 questions about learning process, content, and culture were first retained through factor analysis. A Kaiser-Mayer-Olkin (KMO) test and a Bartlett test for sphericity were performed. The KMO test is an index to measure the applicability of data to factor analysis. It measures the variance ratio of variables [111], and then provides a reference for judging the sufficiency of samples for each variable in factor analysis [112]. The KMO value ranges from 0 to 1, with the value >0.5 acceptable [112]. A significance level of <0.05 for Bartlett’s test of sphericity indicates that the factor analysis is reliable for the data [111]. The result of the KMO test performed by SPSS 25.0 was 0.932. Bartlett’s test for sphericity is significant at the 0.000 significance level. These tests confirmed the reliability and applicability of the variables for factor analysis. Finally, four factors were reduced from 15 questions with a total variance of 75.73%. Accordingly, four factors, namely, F1—information sharing and utilization, F2—management commitment, F3—follow-up, and F4—learning content were extracted (Table 5). 

#### 3.2.2. Exploratory Factor Analysis 

Cronbach’s alpha of the constructs of F1, F2, F3, F4, and safety performance was examined to assess the internal consistency and reliability of scales. The theoretical value of Cronbach’s α varies from 0 to 1, and it greater than 0.7 represents the reliability of the test items is relatively high. Table 6 illustrates that the value Cronbach’s α of each construct was higher than the recommended threshold of 0.700, and confirming the internal consistency of each factor for further analysis. A confirmatory factor analysis was also carried out using AMOS version 24. The factor loading of each measurement index in the confirmatory factor analysis needs to be more than 0.6. Composite reliability (CR) and average variance extracted (AVE) were used to examine convergent validity in this study. The CR for each of the variables was greater than the recommended threshold of 0.700 [113]. Furthermore, the AVE for each of the variables was above the recommended threshold of 0.500 [114]. As shown in Table 6, all constructs used in this study passed the assessment of convergent validity.

### 3.3. Safety Performance Score

**Figure 1:**
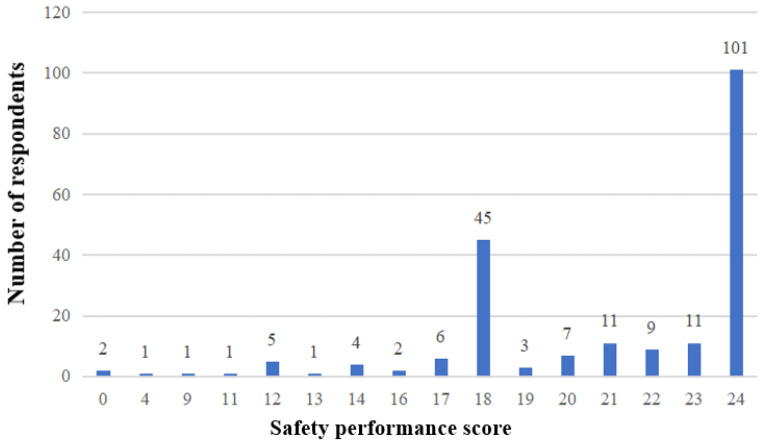
Distribution of safety performance score.

The construct ‘safety performance’ was represented by the overall safety performance score [115]. The score is based on safety performance self-reported by construction workers. The six questions asked the respondents to indicate the level of agreement with the given statement (from strongly disagree to strongly agree). Table 7 shows the cumulative calculation rules for safety performance scores, which theoretically range from 0 to 24. The safety performance scores of construction workers were calculated accordingly, and the results ranged from 0 to 24 (Figure 1). The mean safety performance score of all the frontline workers was 20.95. Use the mean value as a criterion for classifying safety performance [115]. A safety performance scores below the mean value 20.95 was categorised as ‘poor’ safety performance, while a score greater than 20.95 was viewed as ‘good’ safety performance.

### 3.4. Hypothesis Testing (Stepwise Multiple Linear Regression and Model Fitting)

For the causal-effect relationships, the stepwise multiple linear regression was conducted. The independent variables used in this study are the factor scores obtained during the factor analysis process in the previous section. The dependent variable is the safety performance score calculated according to the rules set forth in Table 7. The results of stepwise multiple linear regression shown in Table 8 revealed that all independent variables have a significant impact on the safety performance score, and the differences between safety performance are related to the four factors identified. The correlation coefficient R is 0.839, which reflects the linear correlation between all LFI factors and Safety Performance Score. R Square (R^2^) is 0.704, indicating that 70.4% of the Safety Performance Score variation can be explained by Management commitment, Information sharing and utilization, Follow-up, and Learning content. The closer R^2^ is to 1, the better the model fits the data. The adjusted R^2^ (Adjusted R Square) is 0.698, which is similar to R^2^. It is also one of the important indicators to measure the quality of the model. The larger the adjusted R^2^ value, the better the fitting effect of the model. Stepwise multiple linear regression models the relation between the dependent variable (safety performance score) and independent variables by fitting a linear equation. Table 9 lists the partial regression coefficient (B) of the regression model and its standard error (Std. Error), standardized partial regression coefficient (Beta), t statistics of regression coefficient test, and its *p* value (Sig.). The results showed that the *p* values of the partial regression coefficients of Management commitment, Information sharing and utilization, Follow-up, Learning content, and Constant were all <0.05. At the test level of α = 0.05, it can be considered that the partial regression coefficients are not 0, which is statistically significant and can be included in the final regression model. From Table 9, the relationship between the safety performance score and the four LFI factors can be expressed as:

Safety performance score = 20.952 + 2.723 × Management commitment + 1.743 × Information sharing and utilization +1.006 × Follow-up + 0.838 × Learning content

All identified LFI factors had a significant positive impact on the safety performance score. The regression coefficients of F1-information sharing and utilization, and F2-management commitment are the highest (2.723 and 1.743, respectively).

### 3.5. Bayesian Network (BN) Model 

**Figure 2:**
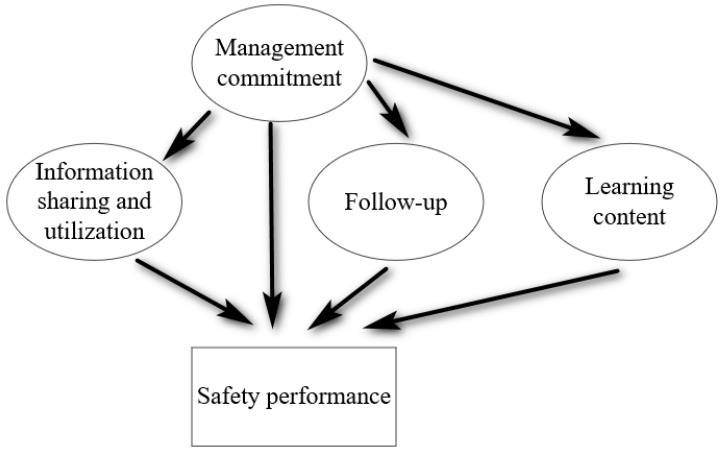
BN structure.

A BN was developed to model the interrelationship between LFI factors and safety performance of frontline workers [115]. Then, the key safety performance influencing factors are identified. Figure 2 illustrates the established BN structure, connections are established amongst ‘information sharing and utilization’, ‘management commitment’, ‘follow-up’, ‘learning content’, and ‘safety performance’. The four identified factors are all direct influences, and ‘management commitment’ is also considered indirect influence.

### 3.6. Network Parameter Learning

The BN structure was previously built on the results of the stepwise multiple regression model and is supported by the literature. The BN calculation process consists of four steps: (1) defining variables, their states, and causation; (2) importing initial data file; (3) setting parameters for all variables in the network (i.e., conditional probability distribution); (4) conducting parameter learning and sensitivity analysis for safety performance inference. The network parameters were further confirmed using the initial data. This process is called parameter learning [116,117]. It aims to determine the conditional probability distribution of each construct based on the established BN structure. Parameter learning is based on Bayes’ theorem and can be expressed by the following formula:

P(A|B) = P(B|A) P(A)/P(B)

The formula states the probability of an event, where P(A|B) is the probability of event A, assuming B occurs; P(B|A) is the probability of event B given that A occurs; P(A) and P(B) are the probability that A and B occur independently. Parameter learning for this study was implemented using NETICA 6.08. Modifications to the developed BN can be made as new information becomes available.

The relationships of the constructs in the BN model were quantified in terms of conditional probabilities calculated from the questionnaire data [115]. During the questionnaire, frontline workers were asked to respond on a five-point Likert scale ranging from “strongly disagree” to “strongly agree.” If each node in the BN were assigned with five states, a large number of samples and a lot of computational work would be required. Therefore, this study first calculated the mean of the items included in each factor [115]. Based on this, the five-point Likert scale was converted into three-point ones using methods suggested by Zhou et al. [118] and Chan et al. [115]. For example:

The states ‘agree’ and ‘strongly agree’ are integrated as ‘good’.

The state of ‘neither disagree nor agree’ is marked as ‘average’.

The states ‘disagree and ‘strongly disagree’ are combined as ‘poor’.

**Figure 3:**
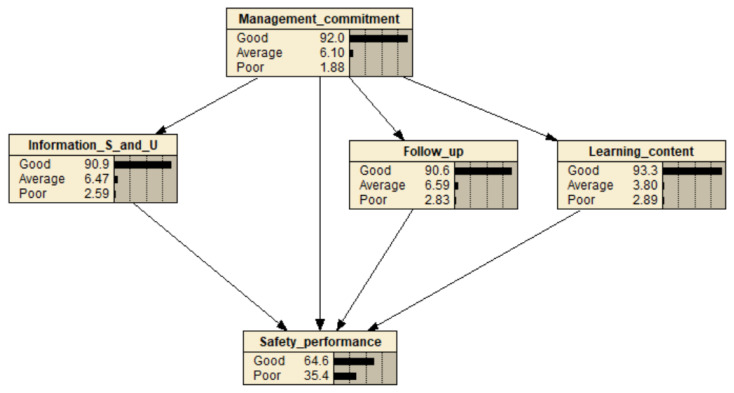
The developed BN model.

The initial data obtained from the questionnaire is used as the input for network parameter learning. The probability of each construct is generated by BN inference. Following the learning of the network parameters, BN can be used as an effective way to estimate the relationships between constructs. Figure 3 illustrates the developed BN model. In the corresponding state, the probability of ‘good safety performance’ was approximately 64.6%, while that of ‘poor safety performance’ was 35.4%.

## 4. Reasoning with the BN (Sensitivity Analysis)

The BN model was established based on the survey data from individual workers in the previous sections. In this section, NETICA 6.08 was used to perform sensitivity analysis of different states of each construct in BN, and estimate the impact of LFI factor changes on safety performance.

### 4.1. Sensitivity Analysis—Single Strategy

Predictive reasoning program can be employed to infer the effect of state changes for each construct on the probability distributions of other sub-constructs. All input LFI factors were analyzed, and the results are shown in Table 10. For example, assuming a management commitment of 100% “Good” in the BN model shown in Figure 3, the probability of “Good” safety performance is expected to change from 64.6% to 66.9% based on the established BN. The probability basically depends on all factors equal to or higher than the parent node of the safety performance node in BN. Accordingly, the single effect can be predicted by observing the change in the percentage of the ‘good’ safety performance of each factor [115]. Table 10 shows that ‘information sharing and utilization’ was the most sensitive to the safety performance amongst all the factors. When the probability of good safety performance rose to 67%, ‘information sharing and utilization’ was rated 100% ‘good’, which indicated a sensitivity of 2.4%. 

Then, the single effect analysis was performed by changing only one factor at a time to the best case in the BN model. Table 11 indicates that probability of good safety performance increased from 41.2% to 66.1% when ‘follow-up’ changed from ‘poor’ to ‘100% good’; ‘management commitment’ changed from ‘poor’ to ‘100% good’, the probability of good safety performance adjusted from 44.6% to 66.9%. The results also showed that ‘learning content’ was the least sensitive factor to safety performance, with a sensitivity of only 7.4%.

### 4.2. Sensitivity Analysis—Joint Strategies

If the better safety performance is required, a joint LFI strategy should be employed when resources are available. This section discusses the results of sensitivity analysis of the joint effect of LFI factors. The results of the sensitivity analysis combining the two different LFI factors are summarized in Table 12. The table shows that the highest probability (68.5%) of “good safety performance” is achieved by combining factors ‘information sharing and utilization = 100% good’ and ‘management commitment = 100% good’. Enhancing “information sharing & utilization” and “management commitment” was the best two-factor strategy to improve safety performance, with the probability of “good safety performance” for construction workers being approximately 68.5%.

## 5. Discussion

Sensitivity analyses can be carried out by the computational program to determine how changes in learning from incident factors (such as management commitment, learning content, information sharing, and utilization and implementation of lessons) impact safety performance. In other words, it can be used to evaluate how the state of each variable affects the probabilities of the other variables. In order to improve safety performance, construction teams can learn from accidents and make better decisions as a result of this analysis. Based on the results of the sensitivity analysis, the top sensitivity nodes were identified, as shown in Table 10. For instance, the likelihood of good safety increased when information sharing and utilization were effective (changing the probability of “Good” state to 100%).

The BN-based approach also assists practitioners in assessing the effects of various strategies and decisions on accident learning in the organization. Practitioners can determine which improvements to organizational learning system elements are the most effective. Controlling the influencing factors of the proposed BN model revealed a higher sensitivity of “Information sharing and utilization” to safety performance. It can be inferred that the “Information sharing and utilization” was very sensitive to safety performance. This was also consistent with previous research, which found that effective accident information sharing and utilization is one of the most important factors influencing safety performance [66,119]. This result is in line with research by Le et al. [82], which found that it is crucial to effectively share and use accident data and safety knowledge in order to lower the likelihood of dangerous accidents happening in the construction sector, as well as its associated risks. An improved accident reporting and communication system is necessary to identify and analyze safety hazards and risk, collect and share incident information, and develop proactive accident prevention methods in the construction process in order to achieve better safety performance [120]. According to Ulang et al. [121], a system for integrating and exchanging information about construction incident knowledge within the construction industry can be established. Workers can obtain the necessary information in a timely manner, share and apply that information to improve their safety knowledge, and make effective decisions [122].

Undoubtedly, effective communication between project managers and supervisory staff and construction workers is essential to ensuring that accident information is conveyed and understood in an accurate and efficient manner [41]. The majority of accident information, according to Azmy [123], is not communicated to on-site workers directly, making it difficult for workers to understand the findings of accident investigations and safety inspections. Azmy [123] suggested that the use of visualization technology allows for the delivery of incident information and lessons learned from on-site incidents and near misses on various construction projects to workers, particularly foreign workers. The development of a web-based communication framework, as suggested by Cheung [124] to manage accident information in construction projects, is one of the applications related to the use of databases in construction safety. The developed system enabled various project safety stakeholders to upload and download information from any location with Internet access. It facilitates the workers to obtain valuable accident or near-miss information in order to improve construction site safety.

Management commitment is also a major factor in improving the safety performance. In the process of reasoning, it was found that “Management commitment” was considered as the second effective factor to increase the probability of a “Good” safety performance. Previous research has shown that management commitment plays an important role in developing a positive safety culture by recognizing incident reporting and learning systems as an essential part of the construction process [125,126,127]. High safety performance was found to require management commitment to learn from incidents [128]. Similar arguments were pointed out by Akson and Hadikusumo [41] and Gao et al. [125], whereby the management commitment is ascertained to influence the ability of the organization to learn from safety, and thus affect the safety performance.

Top management commitment reduces accidents on the job site by fostering a culture of reporting and learning, ultimately improving safety performance. Given that top management’s attitudes toward safety and their investment in safety, such as developing programs for accident reporting and introducing safety training to disseminate incident information, have a significant impact on site level management and frontline workers, it is the most important factor. Lessons learned from accidents were identified in the research of Mohammadi et al. [129] as one of the factors influencing safety performance in the construction industry. In projects or organizations with good safety performance and adequate management commitment to learn from incidents, the managers expressed clearly that incident reporting, investigation was prioritized, and actively share and disseminate incident information between site personnel. According to Hallowell et al. [130], when top management is openly engaged in incident learning, safety performance is exceptionally satisfactory.

In addition to evaluating the sensitivity of individual organizational accident learning factors on safety performance, this section also discusses the sensitivity of the joint effects of factors. Multiple strategies should be used to further improve safety performance if high safety standards are required, and sufficient safety resources are available in an organization. Table 12 demonstrates that “Information sharing and utilization” and “Management commitment” were the most important join strategies. Therefore, changes in the combination of these two factors are important and effective to improve safety performance.

Furthermore, sensitivity analysis of the joint effective revealed that “management commitment” was a component of the three most important joint strategies. The result implied that management commitment plays critical roles in the prevention and mitigation of accidents at construction projects. To encourage a strong safety culture, management support is essential. The organization’s ability to learn from incidents can be improved by management staff support. Management commitment, according to Hong et al. [131], is the foundation of a successful incident learning system. The effectiveness of internal control, and the success of an organization’s incident learning system are both significantly influenced by management’s commitment to safety [126]. To monitor and closely follow-up on the preventive measures on hazards and risks in preventing the occurrence of a workplace accident, a systematic internal control is required. These results suggest that construction companies should concentrate on safety programs to increase management commitment. The key is an organizational commitment that is consistent and observable at all levels.

Implementing lessons is the most important factor that can be used to improve safety performance, according to findings on the influences of factors on safety performance, and comparisons with their worst-case scenarios (Table 11). The likelihood of good safety performance increased from 41.2% to 66.1% when “implementation of lessons” varied from “poor” to “good”. Safety performance has improved by 24.9%. When “management commitment” was changed from ‘poor’ to ‘good,’ the probability of good safety performance increased by 44.6% to 66.9% (a total of 22.3%). The findings indicated that effective “implementation of lesson” is an important part of the incident learning process for reducing the likelihood or risk of an adverse event. It is in line with the research of Zhou et al. [55] that the investigation result of incident and near miss needs to be disseminated to all employees. All involved parties, including employees, production managers, and subcontractors, must be included in the information dissemination process. The daily safety meetings are the main method for informing the workforce about near misses or incidents that have occurred, as well as about the corrective measures that have been planned after these incidents have been analyzed. Foreman and subcontractor representatives participate in weekly meetings for discussing integrated safety and production planning, where information about events helps planning decisions [56]. It is crucial to note that a comprehensive safety information system should include effective dissemination of near-miss or incident information. Other information on safety performance, such as accident reports, unsafe acts and conditions, and information to be aware of at work, should be included in such a system. 

In summary, this research adopted sensitivity analyses to identify the most responsive factors of organizational learning ability from incident. Based on the analysis presented above, predicting the organization’s safety performance becomes more feasible and achievable with the assistance of computational BN programs. The BN program, like the one established for this study, can quickly estimate the risk safety performance, load data directly from spreadsheets, and display and visualize the results. To evaluate the organization’s capacity for learning from accidents and its safety performance, a construction team can input safety data into an Excel template and run the program. Additionally, if the management changed or enhanced specific organizational accident learning factors, the team can forecast the safety performance. The project team may be able to take appropriate action and allocate sufficient resources as a result of these findings to enhance the project’s safety performance.

## 6. Limitations and Future Studies

The current model was developed to learn from incidents involving frontline workers and management personnel in Chinese construction projects. However, as China is the only country included in the study, it would be preferable to compare various LFI with other nations as well. The established BN model served as an illustration. The purpose of the current analysis is to show how processes can be developed and how well LFI factors may be predicted. To improve the safety performance of construction activities, the research outputs are especially helpful to contractors and subcontractors. This analysis also offers insightful information for developing useful suggestions for improving LFI in the future. The created BN model, however, is not a universal computational BN model that can be utilized for any project. Nevertheless, by using the suggested processes, models for estimating the LFI of various projects might be customized. Despite the considerable effort put into this research, some limitations need to be considered for future research. Due to various practical constraints, various factors such as legislation and financial issues and environmental factors were not included in the present study. More survey samples from industrial practitioners will increase the overall credibility of research findings. When new data sources become available, the established BN model can still be further developed. Data from other countries and expert knowledge can be incorporated into the model to enhance its global coverage and accuracy. A similar model can be created for various construction trades.

## 7. Conclusions

A comprehensive understanding of LFI is provided by the literature review on learning from incident factors. Similar instances in the construction business seem to happen frequently, which suggests that LFI is not an effective method there. The effect of LFI on safety performance in construction necessitates a particular strategy to enhance safety performance. For the implementation of a more successful safety management approach, understanding the linkages between LFI becomes essential. The main goal of this study was to identify the effects of the major LFI factors on safety performance of construction workers based on a generic BN model. The significance of this study lies in the proffering of a BN model that reveals the interrelationships of the LFI factors and safety performance of construction works. The findings will help in formulating effective safety management strategies to improve the construction safety. The BN model can be a practical technique to diagnose effective LFI factors for improving safety performance. The research outcomes would be valuable to key project stakeholders to achieve better safety performance, and bring tremendous value in better safeguarding workers’ health and safety. It contributes to provide an updated literature review on the niche area of LFI factors on construction safety. It provides a valid method of analysing LFI factors to derive effective strategies for promoting construction safety. With the help of this useful decision-support tool, industrial practitioners may quickly identify and rank the best methods for minimizing construction accidents. In this study, the structural BN between various LFI components was determined. Based on the opinions of 210 Chinese construction employees, the likelihood of each factor was estimated. According to the BN modeling results, there was a 64.6% chance of achieving “Good” safety performance. The most important element for enhancing a “Good” safety performance was identified by a sensitivity analysis utilizing a BN. The findings demonstrate that “Information sharing and utilization” had the greatest influence on improving a “Good” number of accidents (from 64.6% to 67%). If more resources are available in an organization, multiple strategies can be used to further improve safety performance. The results demonstrate that “Information sharing and utilization” and “Management commitment” were the most important join strategies. Therefore, changes in the combination of these two factors are important and effective to improve safety performance. The findings of this study clearly show how BN modelling and sensitivity analysis can be used to evaluate how different strategies and approaches will affect incident learning within an organization. Practitioners can determine which improvements to organizational learning system elements are the most effective. The findings can be applied to learning from challenging circumstances in other types of projects and other industries, in addition to learning from safety incidents in the construction industry. The methodology can be adopted as an evaluation method as well as a guide for developing holistic organizational learning approaches in a variety of organizations.

## Figures and Tables

**Table 1:** Fall fatality cases in Kai Tak Development project.

	Case Number			
	1	2	3	4
Year	2014	2020	2020	2021
Type of location	On a wooden folding stepladder	On a bamboo scaffold	On ground	On a bamboo scaffold
Type of equipment/structure involved	Stepladder	Bamboo scaffold	Piling equipment	Bamboo scaffold
Type of accident	Fall from ladder	Fall from scaffold	Fall together with the piling equipment	Fall from a bamboo scaffold
Incident Consequences	Death	Death	Death	Death
Accident causes	Improper and unsafe means of support	The upper metal beam was unstably supported using undesirable welded joints	The piling equipment was not placed and operated on firm ground with sufficient load-bearing capacity	Substandard bamboo scaffolds

**Table 2:** Learning from incident factors.

Learning from Incident Factors	References
Question 1. My supervisor would be very helpful if I ask for incident related information.	[83,107]
Question 2. I still receive incident information, even if I am away from site (off shift or on leave).	[83]
Question 3. I immediately inform supervisor, safety practitioner, or workmate once I notice an incident.	[83]
Question 4. I am able to identify my workmate’s or my unsafe behaviours from incident information.	[43,74,108]
Question 5. I modify my unsafe behaviours based on incident information.	[74]
Question 6. I am notified of incident information and corrective measures in a timely manner.	[105]
Question 7. Management and supervisory staff implement appropriate corrective measures immediately following any changes to working conditions (i.e., new hazards identified, hazardous events occurring).	[45,109]
Question 8. At my workplace, there is an atmosphere of trust and openness.	[38,42]
Question 9. Managerial and supervisory staff know how to encourage workers to share their safety experiences.	[43,44,110]
Question 10. I make sure that important information about incidents is shared with others who might benefit from it.	[81,82]
Question 11. At my workplace, workers are informed about the progress and outcomes of incident investigations.	[22]
Question 12. My company implements a reward system that encourages implementation of safety corrective actions.	[24]
Question 13. I participate in incident learning through specialized training program.	[22]
Question 14. I not only learn from previous internal incident experience but also acquire lessons from external sources.	[24,28]
Question 15. At my workplace, I learn from accidents and near misses (an unsafe state in which no damages or injuries actually occurred), regardless of the severity of their outcomes.	[49,56,57]

**Table 3:** The overall research method.

Stages	Process	Aims
1. Developing Questionnaire	1. Develop questionnaire items based on literature review.	To provide a comprehensive understanding of LFI and identify major learning from incident factors.
	2. Conduct pilot study (by six scholars and five construction professionals).	To provide feedback to the questionnaire designed with the aim of ensuring that the statements of the questions could be easily understood by frontline workers.
	3. Revise the questionnaire based on reviewers’ comments.	To refine the questionnaires
2. Data collection	1. Conduct Large-scale questionnaire survey.	To investigate the level of incident learning among frontline workers and management staff in Chinese construction projects.
3. Identifying LFI factors	1. Exploratory Factor Analysis on the 15 questions about learning process, content, and culture.	To identify the underlying dimensions of the LFI factors.
	2. Conduct reliability and validity analysis	To confirm the reliability and applicability of the variables for factor analysis.
4. Hypothesis testing	1. Conduct stepwise multiple linear regression	To determine the effects of the underlying LFI factors on the safety performance of construction workers.
5. Constructing BN model	1. Model the interrelationship between LFI factors and safety performance of frontline workers	To present the probabilistic relationship between LFI factors and safety performance.
	2. Build BN structure based on the results of the stepwise multiple regression model and supported by the literature	
6. Reasoning with the BN	1. Implement parameter learning by using NETICA 6.08	To perform sensitivity analysis of different states of each construct in BN
	2. Perform sensitivity analysis—single strategy	To investigate the impact of major LFI factors on the safety performance of construction workers.
	3. Perform sensitivity analysis—joint strategies	

**Table 4:** Demographic information of 210 frontline workers.

Information about Frontline Workers	Number of Frontline Workers	Percentage (%)
A. Age:		
20 or below	4	1.9%
21–30	61	29.0%
31–40	71	33.8%
41–50	60	28.6%
51–60	13	6.2%
61 or above	1	0.5%
B. Gender:		
Male	187	89.0%
Female	23	11.0%
C. Years of work experience in the construction industry:		
<1 year	20	9.5%
1–5 years	61	29.0%
6–10 years	47	22.4%
11–15 years	30	14.3%
>16 years	52	24.8%
D. Employer type:		
Main contractor	101	48.1%
Subcontractor	82	39.0%
Others	27	12.9%
E. Education level:		
Below primary	2	1.0%
Primary	12	5.7%
Secondary	73	34.8%
Diploma or above	123	58.6%
F. Work experience at current jobsite:		
<1 year	86	41.0%
1–3 years	91	43.3%
>3 years	33	15.7%
G. Current project type:		
Residential building	170	81.0%
Commercial building	15	7.1%
Industrial building	6	2.9%
Infrastructure	7	3.3%
Public building	4	1.9%
Mixed use	7	3.3%
Others	1	0.5%

**Table 5:** The principal component analysis and varimax rotation of LFI factors.

Question	Factor Loading	Eigenvalue	Percentage (%) of Variance Explained	Cumulative (%) of Variance Explained
Factor 1—Information sharing and utilization		8.502	21.436	21.436
Question 1	0.739			
Question 2	0.675			
Question 3	0.643			
Question 4	0.618			
Question 5	0.591			
Question 6	0.568			
Factor 2—Management commitment		1.335	20.807	42.243
Question 7	0.799			
Question 8	0.737			
Question 9	0.722			
Factor 3—Follow-up		0.943	17.835	60.078
Question 10	0.807			
Question 11	0.723			
Question 12	0.645			
Question 13	0.638			
Factor 4—Learning content		0.58	15.653	75.731
Question 14	0.861			
Question 15	0.858			

Note: Factor loading: The correlation coefficients between the variables (rows) and factors (columns); Eigenvalue: The coefficients attached to eigenvectors, which give the amount of variance carried in each principal component; Percentage (%) of variance explained: The value indicates that how much variations in Factor 1–4 are explained by the questions; Cumulative (%) of variance explained: The sum of the sample variances of all individual variables (questions).

**Table 6:** Reliability and validity analysis for constructs in this study.

Constructs	Cronbach’s α	CR	AVE
Factor 1—Information sharing and utilization	0.925	0.928	0.68
Factor 2—Management commitment	0.851	0.809	0.65
Factor 3—Follow-up	0.826	0.742	0.55
Factor 4—Learning input	0.901	0.903	0.82
Safety performance	0.967	0.913	0.83

Note: Cronbach’s α: The Cronbach’s α measures the internal consistency, or reliability, of a set of questions. High Cronbach’s alpha values indicate that response values for each participant across a set of questions are consistent; CR: The Composite Reliability (CR) is a measure of internal consistency in scale items; AVE: The Average Variance Extracted (AVE) is commonly used to validate constructs. AVE is the average amount of variance in observed variables that a latent construct is able to explain.

**Table 7:** Cumulative calculation rules for safety performance scores.

Self-Reported Safety Performance by Frontline Workers	Strongly Disagree	Disagree	Neither Disagree nor Agree	Agree	Strongly Agree
	**1**	**2**	**3**	**4**	**5**
	Safety performance score				
(a) I use all the necessary safety equipment to do my job	0	1	2	3	4
(b) I use the correct safety procedures for carrying out my job	0	5	6	7	8
(c) I ensure the highest levels of safety when I carry out my job	0	9	10	11	12
(d) I promote the safety program within the organization	0	13	14	15	16
(e) I put in extra effort to improve the safety of the workplace	0	17	18	19	20
(f) I voluntarily carry out tasks or activities that help to improve workplace safety	0	21	22	23	24

**Table 8:** Stepwise multiple linear regression model summary.

Model	R	R Square	Adjusted R Square	Std. Error of the Estimate	Sig. F Change
1	0.655 a	0.429	0.426	3.14811	<0.001
2	0.778 b	0.605	0.601	2.62494	<0.001
3	0.815 c	0.664	0.659	2.42834	<0.001
4	0.839 d	0.704	0.698	2.28263	<0.001

Note: a Predictors: (Constant), Management commitment; b Predictors: (Constant), Management commitment, Information sharing and utilization; c Predictors: (Constant), Management commitment, Information sharing and utilization, Follow-up; d Predictors: (Constant), Management commitment, Information sharing and utilization, Follow-up, Learning content.

**Table 9:** Coefficients of the stepwise multiple linear regression model.

Mode		Unstandardized Coefficients		Standardized Coefficients	t	Sig.
		B	Std. Error	Beta		
1	(Constant)	20.952	0.217		96.448	<0.001
	Management_commitment	2.723	0.218	0.655	12.506	<0.001
2	(Constant)	20.952	0.181		115.671	<0.001
	Management_commitment	2.723	0.182	0.655	14.999	<0.001
	Information_sharing_and_utilization	1.743	0.182	0.419	9.601	<0.001
3	(Constant)	20.952	0.168		125.035	<0.001
	Management_commitment	2.723	0.168	0.655	16.213	<0.001
	Information_sharing_and_utilization	1.743	0.168	0.419	10.378	<0.001
	Follow_up	1.006	0.168	0.242	5.989	<0.001
4	(Constant)	20.952	0.158		133.017	<0.001
	Management_commitment	2.723	0.158	0.655	17.248	<0.001
	Information_sharing_and_utilization	1.743	0.158	0.419	11.04	<0.001
	Follow_up	1.006	0.158	0.242	6.372	<0.001
	Learning_content	0.838	0.158	0.201	5.305	<0.001

Note: Dependent Variable is Safety performance.

**Table 10:** Sensitivity of a single factor to improve safety performance.

The Best-Case Probability of a Single Factor	Safety Performance (%)		Sensitivity
	Initial Value *	Updated Value **	
Information sharing and utilization = 100% good	64.6%	67.0%	2.4%
Management commitment = 100% good	64.6%	66.9%	2.3%
Follow-up = 100% good	64.6%	66.1%	1.5%
Learning content = 100% good	64.6%	66.1%	1.5%

* Initial value represents the probability of “good” safety performance (estimated to be 64.6%) when the probability of the other variables remains unchanged. ** Updated value represents the probability of a “good” safety performance when a single factor is changed to the best case and the other three variables are held constant.

**Table 11:** Sensitivity (%) of single factors to improve safety performance.

LFI Factors	Probability at Worst Case		Probability at Best Case		Sensitivity
	State (100%)	Probability	State (100%)	Probability	
Information sharing and utilization	Poor	46.1%	Good	67.0%	20.9%
Management commitment	Poor	44.6%	Good	66.9%	22.3%
Follow-up	Poor	41.2%	Good	66.1%	24.9%
Learning content	Poor	58.7%	Good	66.1%	7.4%

**Table 12:** Sensitivity (%) of the joint strategy to improve safety performance.

The Best-Case Probability of the Joint Strategy	Safety Performance Value (%)		Sensitivity
	Initial Value *	Updated Value **	
Information sharing and utilization = 100% good and Management commitment = 100% good	64.6%	68.5%	3.9%
Follow-up = 100% good and Management commitment = 100% good	64.6%	67.8%	3.2%
Follow-up = 100% good and Information sharing and utilization = 100% good	64.6%	67.1%	2.5%
Learning content = 100% good and Management commitment = 100% good	64.6%	67.8%	3.2%
Learning content = 100% good and Information sharing and utilization = 100% good	64.6%	67.0%	2.4%
Follow-up = 100% good and Learning content = 100% good	64.6%	67.5%	2.9%

* Initial value represents the probability of “good” safety performance (estimated to be 64.6%) when the probability of the other variables remains unchanged. ** The updated value represents the probability of “good” safety performance when the joint strategy becomes the best case and the other three variables remain unchanged.

## Data Availability

Not applicable.
